# At the Epicenter of COVID-19–the Tragic Failure of the Global Supply Chain for Medical Supplies

**DOI:** 10.3389/fpubh.2020.562882

**Published:** 2020-11-24

**Authors:** Sonu Bhaskar, Jeremy Tan, Marcel L. A. M. Bogers, Timo Minssen, Hishamuddin Badaruddin, Simon Israeli-Korn, Henry Chesbrough

**Affiliations:** ^1^Pandemic Health System REsilience PROGRAM (REPROGRAM) Consortium, Innovation & Supply Chain Pandemic REPROGRAM Study Group, Sydney, NSW, Australia; ^2^Department of Neurology & Neurophysiology, Liverpool Hospital and South Western Sydney Local Health District, Liverpool, NSW, Australia; ^3^Neurovascular Imaging Laboratory, Clinical Sciences Stream and NSW Brain Clot Bank, Ingham Institute for Applied Medical Research, Sydney, NSW, Australia; ^4^UNSW Medicine, South Western Sydney Clinical School, The University of New South Wales, Sydney, NSW, Australia; ^5^Innovation, Technology Entrepreneurship and Marketing (ITEM) Group, Eindhoven University of Technology, Eindhoven, Netherlands; ^6^Department of Food and Resource Economics, University of Copenhagen, Copenhagen, Denmark; ^7^Garwood Center for Corporate Innovation, University of California, Berkeley, Berkeley, CA, United States; ^8^Center for Advanced Studies in Biomedical Innovation Law, University of Copenhagen, Copenhagen, Denmark; ^9^Faculty of Law, Lund University, Lund, Sweden; ^10^College of Health and Human Development, Penn State University, State College, PA, United States; ^11^Department of Neurology, Movement Disorders Institute, Sheba Medical Center, Ramat Gan, Israel; ^12^Sackler School of Medicine, Tel Aviv University, Tel Aviv, Israel; ^13^Maire Tecnimont Professor of Open Innovation, Luiss University, Rome, Italy

**Keywords:** coronavirus disease 2019 (COVID-19), blockchain, open innovation, grand challenge, global supply chain, health policy, governance, personal protective equipments (PPEs)

## Abstract

The tragic failure of the global supply chain in the face of the current coronavirus outbreak has caused acute shortages of essential frontline medical devices and personal protective equipment, crushing fear among frontline health workers and causing fundamental concerns about the sustainability of the health system. Much more coordination, integration, and management of global supply chains will be needed to mitigate the impact of the pandemics. This article describes the pressing need to revisit the governance and resilience of the supply chains that amplified the crisis at pandemic scale. We propose a model that profiles critical stockpiles and improves production efficiency through new technologies such as advanced analytics and blockchain. A new governance system that supports intervention by public-health authorities during critical emergencies is central to our recommendation, both in the face of the current crisis and to be better prepared for potential future crises. These reinforcements offer the potential to minimize the compromise of our healthcare workers and health systems due to infection exposure and build capacity toward preparedness and action for a future outbreak.

## Introduction

The coronavirus disease 2019 (COVID-19) pandemic has inflicted severe shortages of acute healthcare materials, equipment, and resources such as personal protective equipments (PPEs), intensive care unit (ICU) beds, hand sanitisers, and mechanical ventilators (1–6). The World Health Organization (WHO) estimates global monthly consumption of 89 million masks, 76 million gloves and 1.6 million goggles (6). This is expected to increase, worsening pressures on health systems that are already under tremendous strain (7, 8). A public health emergency of this scale and scope is unprecedented in developed countries. The tragedy promises to challenge developing countries even more severely in the coming weeks and months. The pervasiveness of COVID-19 globally has exposed that many countries are unprepared and ill-equipped to confront this viral mammoth (9).

There are several initiatives to address the acute need caused by this tragedy. For example, existing manufacturers have expedited the manufacturing process to meet the demands along with many other non-ventilator manufacturers such as Tesla, General Motors, Ford, Dyson and Rolls-Royce, exploring ways to repurpose their existing facilities to manufacture ventilators. Individual user initiatives are also responding to the lack of PPEs in many societies, with online instructions for making one's mask, and homemade recipes for hand sanitizer (3, 4, 10, 11). Despite current initiatives, much more coordination, integration, and management of global supply chains will be needed to mitigate the impact of the pandemics. Below, we first analyze the global supply chain, which has led to the current tragedy, we then propose a new strategy to overcome the pandemics shortfall and finally provide specific policy recommendations concerning regulation and governance of a new supply chain solution. In this paper, we call for a restructuring of governance over the global supply chain in the wake of the critical shortages arising during the COVID-19 pandemic.

## Key Problems With the Current Global Supply Chain in Response to the Pandemic

The global supply chain for critical medical equipment evolved after World War II to reflect multifaceted goals, including broad access, improving quality, and affordability. The large expenditures required to implement technological advances, together with the limited resources of tax-paying patients, has led over time to an intensification of the trade-offs between these goals. The lean supply chain was thus introduced in a bid to improve financial and operational performance. As of now, “lean” management has allowed for more efficient and effective logistical flow as well as improved customer satisfaction (12). But leanness has also led to adversity. Unfortunately, in reducing costs via labor and supply avenues, this has led to a reduction in medical stockpiles which act as “buffers” during crises like COVID-19 (13).

The dominant approach of leanness in managing global supply chains has been accompanied by the implementation of alternative models in several specific countries. For instance, medical units of militaries e.g., the Indian Armed Forces Medical Services, store 6 months' worth of short shelf-life items and 8 months' worth of long-shelf-life items, to create capacity for fast disaster relief (14). Finland, likewise, understood the importance of stockpiling since World War II and kept at it consistently while neighboring Nordic countries like Sweden, Denmark and Norway eventually abandoned their stockpiles (15). Finnish pharmaceutical companies, healthcare units and importers were thus mandated to stockpile medications for up to 3–10 months while being compensated by the government for the cost of preserving such reserves. These models are not without their challenges. Significant variations in supply chain performance arise in individual countries. Some of the existing challenges include lack of a single national procurement unit, disorganized supply chains and most importantly bureaucratic inefficiencies which cause delays across the entire continuum and hence compromise appropriate preparedness for pandemics (13, 16). Information on the current/ongoing efforts is detailed elsewhere (Supplementary Material).

### Low Initial Supplies

Regardless of the supply-chain model, the current global stockpile has been insufficient to support the health system of any country during the pandemic. As of 4 March 2020, the Department of Health and Human Services declared that the United States (US) had ~12 million N95 masks and 30 million surgical masks, making up a mere 1% of the actual required numbers during the pandemic (2). The shortage was exacerbated by the extraction in the US of the 100 million masks held in the national strategic stockpile during the 2009 H1N1 pandemic, as none of these extracted masks were subsequently restocked (17). Similarly, Australia faced a great shortage of masks from the outset of the pandemic. In January 2020, the National Medical Stockpile of Australia was reported to have a stockpile of only 12 million masks (18). However, during the 2009 H1N1 pandemic, the same agency held 40 million masks in reserve (19), implying a lack of restocking since the previous pandemic, more than a decade ago.

### Low Supply From Suppliers

As the virus became pandemic, countries then took steps to protect local supplies (20). China, which normally produces half the global supply of masks at approximately 10 million masks daily, ramped up production to 115 million daily during the early phases of COVID-19 (20). However, the Chinese government simultaneously terminated all mask exports leading to a gradual depletion of global stockpiles. Germany banned the export of the majority of its PPEs (21). In other areas, where local production is not significant, vulnerabilities in the procurement of essential equipment arose. For example, Australia, which imports 90% of its medications, is vulnerable to shortages should supply be impeded (13, 22).

### Sudden Rise in Demand

The exhaustion of PPEs, including masks, and ventilators early in the pandemic led to a rapid plummet in available supply just as an international surge in demand arose in late February and March. The figures are tragic: In 2019, a mere 77,000 ventilators were required globally (11). However, as of 11 March 2020, the US alone required 60,000–160,000 ventilators (4). By mid-April, reports of shortages in critical chemical compounds required to produce essential medicines were rising. The production of other medications might be impeded too, especially if certain required pharmaceutical ingredients can only be sourced from countries that happen to be severely affected by COVID-19. The most important example would be China which exported many raw materials but was temporarily under an economic shutdown. Fortunately, the Food and Drug Administration (FDA) in the US reported shortages of just one drug (22). It is being discussed that the lack of national and public health led cohesive pandemic response and alacrity may have contributed to the rapid increase in cases and high fatality rates.

### Breakdown of Trust Among Supply Chain Stakeholders

The COVID-19 has exposed the fragility of our existing supply chain frameworks. Increasing reports on lack of trust and pressures between various stakeholders have been reported (23). This can be attributed to the presence of middlemen or intermediaries (for contracts and procurements between supplier and buyer), who presumably use opportunistic and unfair business practices underpinning lack of transparency in reporting of stock supply numbers and the ambiguity in movements of transactions. This creates an environment which is fertile for speculation, leading to a breakdown of trust and hence the inter-institutional relationships. In a pandemic setting, this could have disastrous consequences. This is especially poignant as buyers don't believe the data coming from suppliers/middlemen, especially during crises (24, 25).

## Proposal for New Strategies of the Supply Chain to Meet Pandemics Shortfall

**Figure 1:**
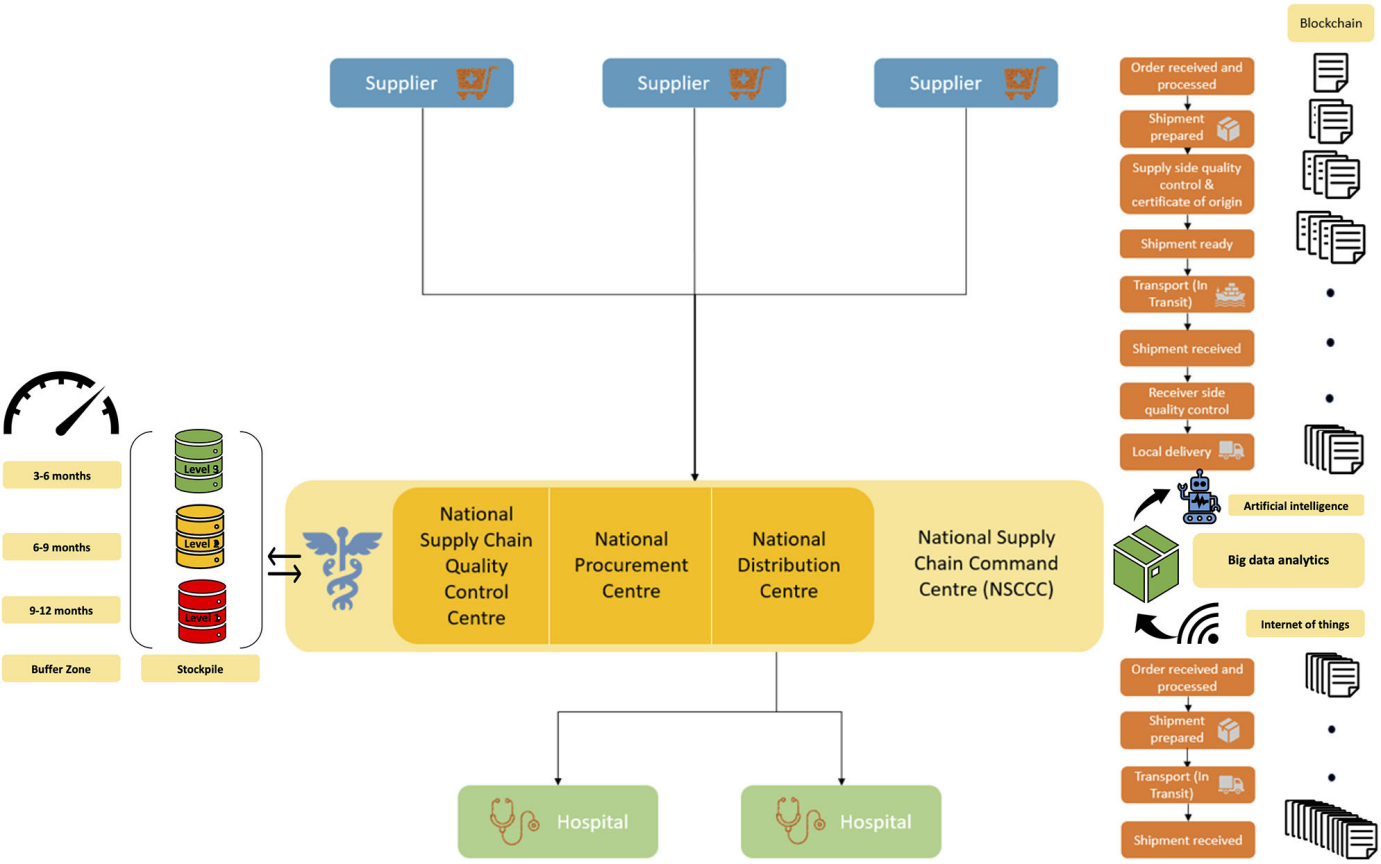
Flowchart of the proposed global supply chain model for healthcare supplies that harnesses blockchain technology as “connector”.

During pandemics, global supply chain systems security and capacity are challenged (26, 27). To this end, technologies such as blockchain, big-data analytics and artificial intelligence could act as enablers toward building robust supply chain models for future (28, 29). In the current proposal, we propose a supply-chain integration framework, that is built around strong governance, minimal bureaucracy and uses technology (e.g., blockchain) as a connector for direct linkages between supply chain stakeholders (buyer and suppliers), that could potentially address the gaps, reduce inefficiencies and build resilient systems. The new proposed supply chain model will optimize inventory and product recall, streamline processes, smoothen procurement and liaison with the suppliers especially the international manufacturers and will provide leadership and accountability in public health crises such as pandemics. It will leverage blockchain technology as connectors between the stakeholders to automate tasks connecting the suppliers directly to the organizations, hence cutting the intermediary while automating audit and reporting on process-related tasks. Hospital-supplier integration has been proven to improve hospital supply chain performance (30). Furthermore, trust enhances the strategic relationship and integration which is mutually beneficial to both hospital and supplier (31). This proposal consists of various elements including governance and organizational structure for supply chain (Figure 1).

**The proposed model consists of the following elements:**

1. **A National Supply Chain Command Centre (NSCCC)** is formed with representations of chief medical officers, independent domain experts (including supply chain, infectious disease, public health, pharmaceuticals etc.), operational experts with appropriate representations from individual states and the federal government. The NSCCC will oversee the procurement and distribution of all health and medical supplies from the country. It will also act as the one-stop command center for regular operational demand and supplies as well as for pandemics or public health emergencies.
2. Under the NSCCC, three arms will be constituted: **National Procurement Centre, National Supply Chain Quality Control Centre and National Distribution Centre**.
3. **The National Procurement Centre** will be responsible for opportunity assessment, engagement, sourcing strategy, tendering, invitation to supply, evaluation and negotiation of contracts, implementation of contracts, and purchase of contracts. It will also be responsible for creating purpose-driven inventory checklists to identify the necessary medical supplies for pandemics. The National Procurement Centre will liaise with the National Supply Quality Control Centre to ascertain needs amidst ongoing and evolving crises such as pandemics.
4. **The National Supply Chain Quality Control Centre** will ensure due diligence in the quality, staged wise stockpiling and appropriate time-buffer for pandemic requirements, and for non-pandemic stocks with pandemic inventory be given high priority and longer buffer/stock period. This stockpile will have three levels of procurement and buffering period: **Level 1** (***Red***) will cater to pandemic needs such as PPEs, surgical and N95 masks, oxygen cylinders, mechanical ventilators and telemedicine capacity including software that can be deployed. There will be a buffer zone of 9–12 months' worth of safety stock. **Level 2 (*Yellow*)** will cater to the supplies of pharmaceuticals and devices inventory that don't have a short expiry period. There will be a buffer zone of 6–9 months' worth of safety stock. **Level 3 (*Green*)** will cater to drugs, medical supplies and devices for non-immediate use. There will be a buffer zone of 3–6 months' worth of safety stock.
5. **The National Distribution Centre** will be the one-stop center that meets the demands of individual hospitals by directly supplying them. This would ensure supply chain consolidation, the economics of scale, efficiency of processes, and optimized distribution. Each hospital will have its own local supply chain operations unit to ensure the local demands of the individual wards and other facilities are met.
6. **Blockchain as “connector”:** In today's world, global supply chains are dynamic where factors such as the life cycle of drugs and medical devices, varying periods of demand surge and decline make the current supply chain complicated. This is further exacerbated in crises such as pandemics (27). Blockchain addresses the global healthcare supply chain from an “ecosystems” approach which provides an integrated view of the elements (29). Blockchain synchronizes data transactions across the network, where each stakeholder can verify the work and calculations. It builds access to reliable, real-time, digital ledger of information on all these transactions across the relevant timepoints (“time and process audit stamp”) in the continuum of the supply chain and process (32). This helps build trust between stakeholders who can access relevant operational details on the supply chain while also allowing stakeholders to negotiate better deals on better terms, cutting down delays between the signing of the contract and product delivery. From an operational and feasibility perspective, it is worthwhile to note blockchain can leverage existing digital infrastructure such as enterprise resource planning (ERP) software, allowing for easy deployment through integration as a single layer onto the existing platform (32, 33). Blockchains can make a huge impact on bringing transparency, improving efficiency and delivery during extended periods of crisis (27). It also cuts down intermediaries and serves as a platform for stakeholders to share data in real-time.
7. **Predictive big-data analytics in supply chain demand forecasting:** The proposed model, built on blockchain technologies, can indeed make use of complementary predictive big-data analytical applications to address future demands through customer behavior analysis, trend analysis, and demand prediction (34). Data collected through the Internet of things (IoT) can also feed real-time data from various sources for big-data analytics (35). The predictive analytics uses various algorithms (e.g., time-series forecasting, support vector machines, K-nearest-neighbors, neural networks) in supply chain demand forecasting to allow the stakeholders to prepare supplies in advance (34). This could also include artificial intelligence platforms toward data-driven proactive demand forecasting which could be useful during infectious disease outbreaks (35). Furthermore, these predictive algorithms can be trained or optimized on the demand and supply data from the current COVID-19 pandemic or previous infection outbreaks such as H5N1 Avian influenza and Ebola outbreaks. Epidemiological studies and modeling should be used to predict the locations of future demand spikes too (36).

The proposed model implements a staged-wise supply chain strategy. Concurrently, manufacturing companies should map out their entire supply chain allowing for more swift decision making. Identifying companies that can produce masks during crises or providing incentives for companies to set up masks production facilities in peacetime that can then be ramped up. Unfortunately, many manufacturers are unwilling to do so due to the perceived large amount of manpower and time (26). Manufacturers should also branch out to more suppliers and consider paying more for backup suppliers in the event of supply disruptions (5).

To mitigate the risk of product expiry, governments may step in to cover the cost of replacing expired products held within stores (37). Ensuring all parts of the “ecosystem” are involved and engaged is important to enabling open innovation across organizational and regional boundaries (38, 39). There needs to be a connection with how much utilization is so that supply can stay ahead of the curve. This can be done through logistics tracking enabled via artificial intelligence (40). Individual users can design, sew and wear their masks, reducing demand on hospital-grade supplies. This is applicable not only for masks but also other critical essential equipment such as ventilators, other PPEs. Some emergency medicine physicians are adapting ventilators to support multiple patients, for example (41–43).

### Regional Stockpiling

Regional stockpiling is another dimension that merits further consideration (44). The Association of Southeast Asian Nations (ASEAN) countries with Japan has made a similar effort previously for avian influenza (45). In the context of COVID-19, the South Asian Association for Regional Cooperation (SAARC) leaders have set-up a COVID-19 emergency fund (46). Such efforts need to be built around a normative framework and institutional arrangements such as the international health framework, created by the WHO's 2005 International Health Regulations (IHR) (47). The 2005 WHO IHR framework pivots around the notion of due diligence (48), the principle of no-harm, and principles of general international law and international human rights law (47). Notwithstanding the limited efficacy and implementation of these initiatives in the past due to regional politics and vested interests, regional approaches to enhance global health security should be intensified (49). We recommend that regional stockpiling infrastructure and framework should be considered and actively pursued for Asia, Middle East, North and South America, Africa and Asia-Pacific regions and that the public-private partnership models be explored.

Eventually, with the proposed streamlined supply chain model, not only will the systems be robust enough to meet demand and supply during pandemics, but the ongoing demands of medical supplies will be appropriately addressed and actioned. This proposal will create a centralized organ that will have proper governance and quality control structures to address future pandemics. Additionally, a hidden army of individuals can contribute user-based designs for masks, gloves, and gowns. Our model would also enable philanthropic groups to identify areas of greatest need and mobilize support for those areas. The public sector must also adapt in these times, to support this initiative. Normal regulatory processes must be accelerated tremendously to get PPEs from this supply chain to those who need it most urgently. If a regulator approves a ventilator for sale in Japan, that approval should have standing in Germany and the US, provided that the approval process and supporting data are provided openly and transparently. Regulators must align their respective procedures and approaches in that regard. Product liability rules that protect consumers should be temporarily relaxed (50), as consumers will be more protected if and when front-line medical staff and other first responders have adequate access to PPEs.

## Policy Implications for Regulation and Governance: the Importance to Embed Rapid Responses Within Sustainable Regulatory Set-Ups

The aforementioned need for accelerated regulatory approval pathways merits considerations of significant accompanying legal implications. Society should be willing to accept somewhat higher risks—if accompanied by transparency and robust informed consents—during this crisis period in return for faster availability of crisis relevant products. But to find more sustainable solutions that protect consumers, patients and healthcare workers, incentivise value-based innovation and enhance global multi-sector collaboration, it is also important to appropriately consider established standards and legal frameworks to the greatest possible extent. While radical emergency responses to ramp up crisis-critical supplies and encourage open innovation can be very nuanced and do not necessarily have to break with rules and traditions of the innovation system (51), such approaches may challenge the legal system as we know it.

Our proposed initiatives must be embedded in the wider legal frameworks and initiatives. These include recent efforts to create voluntary patent pledges and intellectual property (IP) pools, as well as more invasive approaches to increase access to essential technologies through compulsory licensing (52); and competition law (comfort letters by competition authorities, Canada sunset clause, etc.). Yet, many forms of data and know-how protection would often be excluded from these initiatives. We, therefore, call for an international framework for open-source platforms and through international institutions, such as the WHO that also facilitates the Findable, Accessible, Interoperable and Reusable (FAIR) data sharing (53). In the long-term, it will therefore also be important to consider “stick and carrots.” This could include harsh responses by antitrust law to “excessive pricing” and “collusion,” but also fair and reasonable compensation for IPR and data holders, as well as clarity and transparency about both demands and rewards.

Similar considerations must be made with regards to the privacy and data protection implications of the employed technologies, such as blockchain and product tracking or other surveillance systems that could help to stem the spread of coronavirus (54) and facilitate open innovation. This requires finding a balanced middle ground which respects essential privacy principles and democratic rights but also makes sure that overly restrictive data protection rules do not prevent necessary, effective and proportionate measures in the fight against the coronavirus pandemic (55). Lastly, for such an organizational and governance framework to succeed, an autonomous structure is envisioned that's not intimidated by the nation or institutional bureaucracies.

## Conclusion and Recommendations

Current pandemic has exposed the supply chain and responsiveness of the institutions. The COVID-19 has also forced development and adoption of new triage and patient management protocols or pathways to minimize risks to patients, healthcare workers and health systems (7, 56–58), and to maintain patient continuity, using telemedicine, especially to those at high risk of infections or with underlying chronic illnesses (8, 9, 28). The outcry for PPEs especially among healthcare workers, at the forefront of the crisis, warrants overhaul of systems. Political leadership is also being scrutinized and its inefficiencies are getting revealed (59). A robust global supply chain combined with public health strategies and/or interventions such as masks, healthcare worker protection, quarantine, contact tracing, massive testing and travel restrictions could limit rapid COVID-19 spread and build the capacity of our institutions to respond to future pandemics. The army of user contributors should also be crowdsourced and mobilized for this effort.

We suggest that the current “lean” based approaches of the healthcare supply chain model are not appropriate for healthcare and may compromise the economic, global health and national security in crisis such as COVID-19. A scaled open innovation approach, that can provide the buffer to the system should an acute and prolonged need emerge, should be part of future global supply chain systems. This would ensure continued provision of essential healthcare supplies and resilience of the healthcare systems. Technology such as blockchain can act as drivers to further improve the efficiency of supply chains.

Additional efforts and resources will be required to achieve a truly global response that comprises all regions of the world and in particular those which are likely to face the most severe consequences. This requires careful consideration of regional disparities in terms of economic capacities, technical infrastructures and cultures. Most importantly, to be better prepared for the next pandemic it will be important to learn lessons from the current COVID-19 crisis to improve global fast-track emergency procedures and global stockpiles with several suppliers on a more sustainable level. Building on the proposed supply chain model, aspects of integration and implementation of national public health policies would also need to be considered. Ideally, the processes that lead to these solutions should be characterized by more effective interdisciplinary collaboration, evidence-informed decision-making, proportionality, flexibility, precautionary approaches combined with established practices, improved regulatory frameworks and ethical decision-making. Good governance, openness and collaboration will be key to effectively fast-track responses–even in the next pandemic. It is therefore important that time-limited radical responses, required right now, will be re-evaluated when the situation improves, to achieve a more dynamic and adaptable but still sustainable model in times of crisis.

The COVID-19 is undoubtedly a public health crisis of a scale not witnessed in a century (60). Fears of impending economic crises reminiscent of the “great depression” abound. Should the economic doomsday predictions come true, the already challenged global health status could take a downward spiral with long-term implications for health and well-being of people (61). Therefore, a crisis like this warrants an unprecedented global effort. Lukewarm responses of governments and health care institutions in determining what work is essential and what the correct PPE is for each situation has caused the build-up of anxiety, confusion, possible increased viral transmission and misuse of PPE (62). Public awareness campaigns on the need to use masks and gloves have also been confusing. As a result of the confusion, the public and healthcare staffs are generally left to look after themselves and use their own. Therefore, clear public health and occupational safety policies and guidelines should be developed for implementation right from the hospital level to the national level to increase public and healthcare workers' adherence to best practices in adherence to infection control including the practice of wearing PPEs, hand-washing and social-distancing (63). Coupled with a lack of regulation, this has seen a surge in the number of “intermediaries” buying and selling PPEs. Moreover, existing “lean” based approaches of healthcare supply chain models have resulted in a shortage of PPEs, possibly compromising economic, health and national security.

In the advent of a lock-down and strained global supply chains, the lack of policies or frameworks to ensure the limited PPEs reach those who need it most (healthcare workers) has caused an outcry, warranting an overhaul of existing systems. Perhaps by enforcing such regulation, that guarantees the provision of critical supplies to protect healthcare workers while they continue to treat patients, the deaths of healthcare workers could be avoided (64–66). We thus call for an international body such as the United Nations to create and implement the proposed framework through which private- and public-sector institutions analyze, restructure and implement new systems to ensure that health-system resilience is no longer compromised by the failure of global supply chains. Such a body would be complementary to the WHO and would amplify its impact. The COVID-19 Technology Access Pool (C-TAP) launched by the WHO, aimed at equitable distribution of medical supplies, can also be a potential solution (67). The C-TAP initiative would allow worldwide sharing of patents covering pharmaceuticals, vaccines, and/or methods of treatment related to the COVID-19. Call for waiver of IP rights to World Trade Organization, by countries like South Africa and India, to enable widening of, and ease of, access to, COVID-19 drugs, diagnostics and vaccines, especially by low-income countries is a promising development toward ensuring equitable access (68). European Commission has also released antitrust guidance to allow limited cooperation among companies, concerning critical hospital medicine shortages during the COVID-19 outbreak (69).

A robust global supply chain combined with public health strategies and/or interventions such as contact tracing would build the capacity of our institutions to respond to future pandemics. In this way, we will not only be able to address the urgent needs across the world—not the least in developing countries—but also be better prepared for the next pandemic.

## Limitations

We acknowledge that the real-world implementation of the model proposed in this article and the global coordination depends on several factors including good relations between the participating countries which is subject to several geopolitical and diplomatic considerations. Given the current geopolitical situation, its feasibility for a global roll-out may be challenging. However, we believe the implementation should be pursued in a stage-wise plan with the initial implementation, to begin with, to involve countries more amenable to collaboration. This requires to enable openness and collaboration across levels of analysis, for example, not only across organizations but also on an international level (38). Nevertheless, the modular nature of the proposed supply chain model makes it appropriate for scaling-up, including at a global level. Furthermore, a pilot implementation would also provide data for further improvement in improving the system's workflows.

## Data Availability Statement

The original contributions presented in the study are included in the article/Supplementary Materials, further inquiries can be directed to the corresponding authors.

## Author's Note

The COVID-19 pandemic is causing an unprecedented public health crisis impacting healthcare systems, healthcare workers, and communities. The COVID-19 Pandemic Health System REsilience PROGRAM (REPROGRAM) consortium is formed to champion the safety of healthcare workers, policy development, and advocacy for global pandemic preparedness and action.

## Author Contributions

SB devised the project, the main conceptual ideas, including the proposal for the blockchain-based supply chain model, the proof outline, and coordinated the writing and editing of the manuscript. SB and JT wrote the first draft of the manuscript. SB encouraged JT to investigate and supervised the findings of this work. All authors discussed the results and recommendations and contributed to the final manuscript. This paper could only consider developments until April 30, 2020, at the time of manuscript writing and submission. However, some updates were considered and added during the revision of the manuscript in October 2020. It can, however, be expected that some of these initiatives will meet reluctance or outright opposition from major industry stakeholders. The opinions expressed in this article are those of the authors and do not necessarily represent the decisions, official policy or opinions of the affiliated institutions.

## Conflict of Interest

The authors declare that the research was conducted in the absence of any commercial or financial relationships that could be construed as a potential conflict of interest.
