# Supplementary material for: At the Epicenter of COVID-19–the Tragic Failure of the Global Supply Chain for Medical Supplies
Source: Front Public Health. 2020 Nov 24;8:562882. doi: 10.3389/fpubh.2020.562882 (PMC7737425; doi:10.3389/fpubh.2020.562882)
Supplement: Supplementary file 1 [file Data_Sheet_1.PDF]

SUPPLEMENTARY INFORMATION

<u>Full Title:</u> **At the Epicentre of COVID-19 - the Tragic Failure of the Global Supply Chain for Medical Supplies**

<u>Short Title:</u> **COVID-19 and Medical Supply Chain**

Authors: Sonu Bhaskar, Jeremy Tan, Marcel Bogers, Timo Minssen, Hishamuddin Badaruddin, Simon Israeli-Korn, Henry Chesbrough

Current efforts

Several non-healthcare related companies such as Ford and Dyson have begun manufacturing ventilators, masks and 3D-printed face shields to help relieve severe shortages of PPE [1]. General Motors has begun working with Ventec to produce more ventilators by sharing their expertise. Medtronic produced the ventilator PB 560 and even offered up its design specifications freely to any manufacturer in hopes that, by pooling resources together, more can be done to overcome the gaping hole of medical supplies [2]. Additionally, other alternatives include considering ambulance respirators and hand pumps [1]. Apart from this, more draconian measures resemble the US' invoking of the Defence Production Act to redirect existing resources to specific states. The US asked Minnesota-based company 3M to redirect shipment of 10 million N95 masks produced in Singapore for distribution to Healthcare workers in Asia back to the US [3].

India is creating ICU beds on repurposed trains for catering to COVID-19 cases [4]. The country has started a national campaign for people to create home-made masks with easy do it yourself (DIY) posters being shared with people through various communication channels including social media, television broadcast, messaging apps and through community-based volunteers [5]. This exemplifies how crowdsourcing interventions and frugal innovations can be a great strategy for public health interventions in under-resourced and large population settings.

Asian countries such as South Korea have taken proactive and innovative strategies at the very onset of the Coronavirus crisis. The premise being that early intervention is key to

limiting the extent of the contagion and to prevent deaths. Aggressive infectious disease control was used such as tracking, enforced quarantines and nationwide social distancing measures. These were effective due to the alacrity and transparency with which the respective political and health system leadership in these countries responded to contain SARS-CoV-2 [6]. It is pertinent that the lessons learned from the SARS epidemic may have contributed towards a relatively successful public health response from these countries [7].

Due to her former experience with SARS in 2003, Singapore was prepared with stockpiled masks. Apart from the Singapore government advising only the sick to wear surgical masks, 5 million masks were distributed to 1.37 million households in February [8]. In April, the Singapore government went on to distribute reusable masks to every individual [9]. Likewise, since the adoption of a national pandemic response plan after the SARS epidemic, Taiwan has shown capabilities in combatting a pandemic like COVID-19 with an initial 44 million surgical masks, 1.9 million N95 masks and 1,100 negative pressure isolation rooms while continuing production of 10 million masks daily [10].

Proactive travel restrictions were also imposed early (February 1, 2020) by Taiwan, Hong Kong and Singapore for incoming passengers from China in contravention of then existing WHO advisory which didn’t mandate travel restrictions. Despite the major economic implications and cost to these countries, the political leadership deserves commendation for rapid and effective measures. Subsequent to the SARS epidemic, Taiwan established the National Health Command Center (NHCC) focussing on large-outbreak response for pandemics. The NHCC in Taiwan identified 124 action items including border control, assessment of hospital resources, public communication plans, quarantine of suspected cases, etc. [11].

Supplementary References

[1] T. Netland, A better answer to the ventilator shortage as the pandemic rages on. *World Economic Forum* (2020), (available at https://www.weforum.org/agenda/2020/04/covid-19-ventilator-shortage-manufacturing-solution/).

[2] Medtronic Shares Ventilation Design Specifications to Accelerate Efforts to Increase Global Ventilator Production. *Medtronic* (2020), (available at http://newsroom.medtronic.com/news-releases/news-release-details/medtronic-shares-ventilation-design-specifications-accelerate).

[3] J. Politi, D. Sevastopulo, L. Fedor, A. Edgecliffe-Johnson, White House tried to force 3M to send masks from Singapore to US. *Ft* (2020), (available at https://www.ft.com/content/cee34681-5f47-416b-9cbc-d824e9eec68e).

[4] H. Ramaprasad, India has closed its railways for the first time in 167 years. Now trains are being turned into hospitals. *CNN* (2020), (available at https://edition.cnn.com/2020/04/05/asia/indian-railways-coronavirus-hospitals-intl-hnk/index.html).

[5] Virus Crisis: After WHO & US, India Urges Outdoor Use of Masks, Releases DIY Guide for 'Face Covers'. *News18* (2020), (available at https://www.news18.com/news/india/coronavirus-pandemic-after-who-us-india-urges-use-of-masks-outdoors-releases-diy-guide-for-face-covers-2564211.html).

[6] H. Beech, Tracking the Coronavirus: How Crowded Asian Cities Tackled an Epidemic. *Nytimes* (2020), (available at https://www.nytimes.com/2020/03/17/world/asia/coronavirus-singapore-hong-kong-taiwan.html).

[7] L. Barron, Coronavirus Lessons From Singapore, Taiwan and Hong Kong. *Time* (2020), (available at https://time.com/5802293/coronavirus-covid19-singapore-hong-kong-taiwan/).

[8] S. Khalik, Wuhan virus: Every household in Singapore to get 4 masks; collection starts on Feb 1. *The Straits Times* (2020), (available at https://www.straitstimes.com/singapore/wuhan-virus-every-household-in-singapore-to-get-4-masks-collection-starts-on-feb-1).

[9] COVID-19: Safety measures in place for reusable mask distribution from Apr 5. *CNA* (2020), (available at https://www.channelnewsasia.com/news/singapore/covid-19-safety-measures-reusable-mask-distribution-coronavirus-12609692).

[10] S. Chen, Taiwan sets an example for the world on how to fight coronavirus. *ABC News* (2020), (available at https://abcnews.go.com/Health/taiwan-sets-world-fight-coronavirus/story?id=69552462).

[11] C. Wang, C. Ng, R. Brook, Response to COVID-19 in Taiwan - Big Data Analytics, New Technology, and Proactive Testing. *JAMA.* **323**, 1341-1342 (2020). DOI:10.1001/jama.2020.3151
